# Epigenetics in diagnosis of colorectal cancer

**Published:** 2016-03

**Authors:** Aga Syed Sameer, Saniya Nissar

**Affiliations:** 1 Basic Medical Sciences Department, College of Medicine, King Saud bin Abdulaziz University for Health Sciences, Jeddah, Kingdom of Saudi Arabia; 2 Department of Biochemistry*,* Kashmir University, Hazratbal, Srinagar, Kashmir, INDIA

**Keywords:** olorectal Cancer, Epigenetics, Hypermethylation, Biomarkers

## Abstract

Colorectal cancer (CRC) is a third most common epithelial carcinoma. CRC is known to develop from the early precancerous lesion to full blown malignancy via definite phases due to cumulative mutations and aberrant methylation of number of genes. The use of serum biomarkers that is non-invasive to discriminate cancer patients from healthy persons will prove to be an important tool to improve the early diagnosis of CRC. This will serve as the boon to the clinical management of the disease.

## INTRODUCTION

Colorectal cancer (CRC) is a multifactorial disease that arises due to the cumulative accumulation of genetics as well as epigenetic alterations in a number of onco-, tumor suppressor-, mismatch repair-, cell cycle- genes in colon mucosa cells [1, 2]. All of these alterations aggregate to drive the critical pathways of CRC initiation and progression along a multistep tumorigenesis process, known as the adenoma-carcinoma sequence or vogelgram [3, 4]. Since CRC has been defined as the heterogenous malignancy, it has several different subtypes, each of which are characterized by distinct genetic, cytogenetic and epigenetic alterations [1, 2, 5, 6]. A number of specific phenotypes of CRC have been identified on the basis of molecular profiles as given by Vogelstein et al., [3] and defined as the genetic instability model of colorectal carcinogenesis. Two major mechanisms of genomic instability in CRC have been given for the evolution of normal mucosa to adenoma and carcinoma namely: chromosomal instability (CIN), microsatellite instability (MSI) [5, 7]. However, a decade of elucidating the alternative mechanism of colorectal carcinogenesis since the discovery of high frequency of aberrant DNA methylation in CpG islands of number of genes by Toyota et al., [8, 9] and a recent identification of a number of genes that are more frequently methylated in CRC by Lao and Grady [10], epigenetics has been identified as one of the important pathways for CRC carcinogenesis [11] to explain the transformation of the normal mucosa into the malignant one [12]. Hence, CpG island methylator phenotype (CIMP) has been added as the third mechanism for driving the colorectal carcinogenesis [1, 5, 6, 13, 14]. In this minireview, we are aiming to understand the diagnostic value of the methylated genes in serving as biomarkers for early as well as advanced stage CRC detection.

**DNA methylation in Diagnostics**

As DNA methylation plays a significant role in CRC initiation it helps in identifying the strong biomarkers (as methylated DNA) for the early detection of CRC. Many studies have identified a number of genes which serve as the potential biomarkers for the CRC [15-20, see Table 1]. The reported sensitivities for blood and stool based CRC DNA methylation biomarkers range in between 90-95% with a specificity ranges of 85-94% [21]. Among the most potential diagnostic biomarkers for CRC, the tumor specific M2 isoform of pyruvate kinase (*PKM2*) and tissue inhibitor of matrix metalloproteinase 1 (*TIMP1*), vimentin (*VIM*) and septin 9 (*SEPT9*) take a lead in being the most extensively investigated ones [17]. *PKM2* has been shown to have a relatively high sensitivity for CRC diagnosis, with sensitivity of over 90% and *TIMP1* of 63% in stool for CRC [22, 23]. *SEPT9* has been reported to have higher sensitivity (80%-90%) and specificity (80%) [24-26] while as *VIM* has a sensitivity ranging from 38–88 % [18]. Both *SEPT9* and *VIM* are available in commercially available kits and are widely used for the detection of CRC [18, 24, 27].Warren et al., [25] had also identified *SEPT9* as the specific blood based biomarker for the detection of CRC with the overall sensitivity of 90%. Church et al., [28] also reported the similar observations for the accuracy of circulating methylated *SEPT9* DNA to detect CRC, with the sensitivity of 48.2% and 91.5% specificity. A recent study by Carmona et al., [16] observed the potentiality of five selected genes *VIM*, *SEPT9*, angiotensin II receptor, type 1 (*AGTR1*), wingless-type MMTV integration site family member 2 (*WNT2*), to serve as the biomarkers for non-invasive early detection of colorectal cancer using stool DNA (sDNA). In this study three of five selected genes i.e., *AGTR1*, *WNT2* and slit homolog 2 (*SLIT2*) were validated in stool DNA of affected patients with a detection sensitivity of 78% [95% confidence interval (CI), 56%–89%]; while as, DNA methylation of *VIM* and *SEPT9 *was evaluated in a subset of stool samples yielding sensitivities of 55% and 20%, respectively. Thus, indicating that sDNA test achieved greater sensitivity than SEPT9. A recent study by Ahlquist et al., [26] reported a DNA stool test that detects methylated Bone morphogenic protein 3 (*BMP3*), NDRG family member 4 (*NDRG4*), *VIM* and tissue factor pathway inhibitor 2 (*TFPI2*), mutant *KRAS*, the actin beta (*ACTB*) gene and the quantity of hemoglobin. Lee et al., [29] identified another set of biomarkers i.e., O-6-methylguanine-DNA methyltransferase (*MGMT*), Ras association (RalGDS/AF-6) domain family member 2 (*RASSF2A*), and Wnt inhibitory factor 1(*Wif-1*) genes for the early detection of CRC. Another study by Wasserkort et al., [30] also corroborated that *SEPT9* is an aberrantly hypermethylated in one of several CpG islands in adenoma and CRC specimens reflect the cellular progression towards malignancy in colon mucosa.

**Table 1:** Various methylated genes used as potential biomarkers for the detection of CRC

**Study**	** Genes methylated in CRC**	**Biomarker in**
Carmona et al^16^	*VIM*, *SEPT9*, *AGTR1*, *WNT2*	Stool
Fung et al^17^	*PKM2*, *VIM*, *TIMP1*, *SEPT9*	Serum/Plasma
Kim et al. ^19^	*ADHFE1*, *BOLL*, *SLC6A15*, *ADAMTS5*, *TFPI2*, *EYA4*, *NPY* TWIST1,LAMA1,GAS7,MAEL,SFT2D3	Tissue
Ahlquist et al^26^	*BMP3*, *NDRG4*, *VIM*, *TFPI*	Stool
Lee et al^29^	*MGMT*, *RASSF2A*, *Wif-1*	Plasma
Wasserkort et al^30^	*SEPT9*	Tissue
Imperiale et al.^31^	*BMP3*, *NDRG4*	Stool
Melotte et al.^32^	*NDRG4*	Stool
Silva et al^36^	*RUNX3*, *PCDH1*, *SFRP5*, *IGF*, *Hnf1b*	Tissue
Ogino et al.^37^	*RUNX3*, *CACNA1G*, *IGF2*, *MLH1*	Tissue
Wallner et al.^38^	*HLTF*, *HPP1*/*TPEF*, *hMLH*	Serum
Philipp et al.^39^	*HLTF*, *HPP1*	Serum
Tanzer et al^40^	*ALX4*, *SEPT9*	Serum
Lofton-Day et al^41^	*SEPT9*, *TMEFF2*, *NGFR*	Plasma
Vedeled et al^42^	*DLCK1*	Tissue
Mitchell et al.^43^	*SOX21*, *SLC6A1*, *NPY*, *GRASP*, *ST8SIA1*, *ZSCAN18*	Stool
Mitchell et al.^43^	*BCAT1*, *COL4A2*, *DLX5*, *FGF5*, *FOXF1*, *FOXI2*, *GRASP*, *IKZF1*, *IRF4* SDC2 and SOX21	Blood
Roperch et al.^44^	*NPY*, *PENK*, *WIF1*	Tissue and serum
Ahn et al.^45^	*WNT5A*,*SFRP1*, *SFRP2*, *hMLH1*, *p16*, *p14*, *MINT1*, *MINT2*, *MINT31*	Tissue
Lind et al.^ 46^	*CNIP1*, *FBN1*, *INA*, *SNCA*, *MAL*, *SPG20*	Tissue

In this year’s pioneer study by Imperiale et al., [31] a noninvasive, multitarget stool DNA test was compared with fecal immunochemical test (FIT) for the detection of colorectal cancer. It was observed by them, that multitarget stool DNA testing detected significantly more cancers than did FIT but had more false positive results, with sensitivity for detecting CRC was 92.3% using stool DNA testing and 73.8% with FIT (P = 0.002). The multitarget stool DNA test consists of molecular assays for aberrantly methylated *BMP3* and *NDRG4* promoter regions, mutant KRAS, and β-actin (a reference gene for human DNA quantity), as well as an immunochemical assay for human hemoglobin. The study by Melotte et al., [32] had the similar results with another gene namely NDRG4. They found that a significant promoter hypermethylation of NDRG4 promoter in CRC tissue when compared to normal colon tissue, and hence identified NDRG4 as a potential CRC biomarker in stool. Furthermore a number of studies identified a Kunitz-type serine proteinase inhibitor, namely *TFPI2*, as a potential sDNA marker as well as a prognostic marker for CRC [33-35]. 

In another study by Silva et al. [36], a group of five genes i.e., runt-related transcription factor 3 (*RUNX3*), protocadherin 10 (*PCDH10*), secreted frizzled-related protein 5 (*SFRP5*), insulin-like growth factor 2 (*IGF2*) and hepatocyte nuclear factor 1β (*Hnf1b*) were found to be having highest percentage of methylation within their promoter regions and consequently with highest repression of gene expression in CRC patients. Hence, they were identified to be, therefore, the most promising biomarkers for the diagnosis of CRC. Ogino et al., [37] had previously identified the panel of eight genes: *RUNX3*, calcium channel, voltage-dependent, T type, alpha 1G subunit (*CACNA1G*), *IGF2*, mutL homolog 1 (*MLH1*), neurogenin 1 (*NEUROG1*), cellular retinoic acid binding protein 1 (*CRABP1*), suppressor of cytokine signaling 1 (*SOCS1*), and cyclin-dependent kinase inhibitor 2A (*CDKN2A*) out of which at least four (*RUNX3*, *CACNA1G*, *IGF2*, and *MLH1*) were identified to serve as a sensitive and specific marker panel for CIMP-high. 

Wallner et al., [38] in their multivariate analysis identified three methylation markers: helicase-like transcription factor (*HLTF*), hyperpigmentation, progressive 1/transmembrane protein containing epidermal growth factor and follistatin domain (*HPP1*/*TPEF*), and *hMLH *in serum of colorectal cancer patients to be independently associated with poor outcome and a relative risk of death. Hence, these genes were identified as pre-therapeutic predictor of the outcome of disease. A separate study by Philipp et al., [39] reported that the methylation of *HLTF* and *HPP1* DNA in serum was significantly associated with tumor size, stage, grade and metastatic disease and hence were identified as independent prognostic factors in metastasized CRC. Tanzer et al., [40] observed that serum methylated DNA from advanced precancerous colorectal lesions can be detected using a panel of two DNA methylation markers, aristaless-like homeobox 4 (*ALX4*) and *SEPT9*. They observed a significantly higher frequency of *ALX4* and *SEPT9* methylated DNA in plasma from patients with polyps as well as colorectal adenomas versus healthy controls. Both these markers had a sensitivity and specificity of 71% and 95%, respectively, for the detection of advanced precancerous colorectal lesions. Another pioneer study on the blood-based detection of methylated DNA by Lofton-Day et al., [41] identified three genes: *SEPT9*, transmembrane protein with EGF-like and two follistatin-like domains 2 (*TMEFF2*) and nerve growth factor receptor (*NGFR*), to serve as sensitive biomarkers for CRC. *SEPT9*, methylation was detected in 69% of plasma samples from CRC patients, while as *TMEFF2* and *NGFR* methylation status were 65% and 51% respectively.

Another study by Vedeled et al., [42] identified a new gene double cortin-like kinase 1 (*DLCK1*) promoter hypermethylation as a promising new novel epigenetic biomarker for early detection of CRC. They observed a significant negative correlation between *DCLK1* methylation pattern and expression in 74 cancer cell lines derived from 15 different tissues. The gene expression also showed a direct correlation with epigenetic drug induced silencing which increased significantly after drug treatment of initially methylated cancer cell lines. However the testing being invasive has certain limitation in putting it into practice. Mitchell et al., [43] identified a panel of 23 genes that show elevated DNA methylation in >50% of CRC tissue in comparison to non-cancerous tissue. These 23 genes consisted of collagen type 1 alpha 1 (*COL1A2*), collagen type IV alpha 1 (*COL4A1*) Collagen type IV alpha 2 (*COL4A2*), distal-less homeobox 5 (*DLX5*), EGF-like repeats and discoidin I-like domains 3 (*EDIL3*), EGF containing fibulin-like extracellular matrix protein 2 (*EFEMP*), Fibrillin 1 (*FBN1*), fibroblast growth factor 5 (*FGF5*), forkhead box B1 (*FOXB1*), forkhead box D2 (*FOXD2*), forkhead box F1 (*FOXF1*), general receptor for phosphoinositides 1 (*GRASP*), iroquois-related homeobox 1  (*IRX1*), meis homeobox 1 (*MEIS1*), matrix metallopeptidase 2 (*MMP2*), neuropeptide Y gene (*NPY*), pancreatic and duodenal homeobox 1 (*PDX1*), protein phosphatase 1 regulatory inhibitor subunit 14A (*PPP1R14A*), syndecan 2 (*SDC2*), Sry-related HMG box 21 (*SOX21*), sushi domain containing 5 (*SUSD5*), transcription factor 21 (*TCF21*) and Zinc Finger Protein 471 (ZNF471). Out of 23, six genes (*SOX21*, solute carrier family 6 member 15 (*SLC6A15*), *NPY*, *GRASP*, sialyltransferase 8 (alpha-N-acetylneuraminate: alpha-2,8-sialytransferase, *GD3* synthase A1 (*ST8SIA1*) and zinc finger & SCAN domain containing 18 (*ZSCAN18*) show very low methylation in non-cancerous colorectal tissue and hence were identified as candidate biomarkers for stool-based assays, while as 11 genes (branched chain amino-acid transaminase 1 (*BCAT1*), *COL4A2*, *DLX5*, *FGF5*, *FOXF1*, *FOXI2*, *GRASP*, ikaros family zinc finger protein1 (*IKZF1*), interferon regulatory factor 4 (*IRF4*), *SDC2* and *SOX21* have very low methylation in peripheral blood DNA and hence were suitable for blood-based diagnostic markers as these 11 genes were found to be hypermethylated in at least 70% of cancerous tissues. Roperch et al., [44] observed that *NPY*, proenkephalin (*PENK*), and Wnt inhibitory factor 1 (*WIF1*) can be used as combined epigenetic markers for the diagnosis of CRC, both in tissue and serum. Ahn et al., [45] identified wingless-type MMTV integration site family, member 5A (*WNT5A*), secreted frizzled-related protein 1 (*SFRP1*), secreted frizzled-related protein 2 (*SFRP2*), human mutL homolog 1 (*hMLH1*), *p16*, *p14*, methylated in tumor (*MINT1*, *MINT2*, and *MINT31*) to be good prognostic markers of CIMP+ CRC. Lind et al., [46] identified six different highly sensitive and specific biomarkers *CNIP1*, *FBN1*, internexin neuronal intermediate filament protein, alpha (*INA*), synuclein alpha SNCA, myelin and lymphocyte protein gene (*MAL*), and *SPG20*, all of which displayed significantly higher methylation pattern in both adenomas and carcinomas. In addition co-methylation of all six genes was detected in 99% of CRC samples and in 90 % of adenomas. 

Kim et al., [19] have also identified ten gene biomarkers: alcohol dehydrogenase, iron containing 1 (*ADHFE1*), Boule-like RNA-binding protein (*BOLL*), *SLC6A15*, *ADAM* metallopeptidase with thrombospondin type 1 motif 5 (*ADAMTS5*), *TFPI2*, eyes absent homolog 4 (*EYA4*), *NPY*, twist family BHLH transcription factor 1 (*TWIST1*), laminin, alpha 1 (*LAMA1*), and growth arrest-specific 7 (*GAS7*) and two genes: maelstrom spermatogenic transposon silencer (*MAEL*), SFT2 domain containing 3 (*SFT2D3*) showing hypomethylation in CRC tissues. The study analyzed the methylation profile of 27,578 CpG sites spanning more than 14,000 genes in CRC.

In conclusion, the focus of this article was on the use of various methylated genes to be used as the diagnostic markers for the early detection of CRC for the better clinical management of the disease. Currently, fecal occult blood test (FOBT) is the only screening modality used for the detection of CRC together with CEA to monitor the therapy in advanced CRC. A number of companies working in junction with FDA are now focusing to utilize the sensitivity of many methylation sensitive genes like *SEPT9*, *TIMP-1*, *NGFR* to be used as early markers with a simple blood testing kits available over the counter for clinical use [47, 48]. 
